# Supplementary material for: Moisture Resistance, Thermal Stability and Fire Behavior of Unsaturated Polyester Resin Modified with L-histidinium Dihydrogen Phosphate-Phosphoric Acid
Source: Molecules. 2021 Feb 10;26(4):932. doi: 10.3390/molecules26040932 (PMC7916693; doi:10.3390/molecules26040932)
Supplement: Supplementary file 1 [file molecules-26-00932-s001.pdf]

molecules

MDPI

Supporting information

Article

Moisture Resistance, Thermal Stability and Fire Behavior of Unsaturated Polyester Resin Modified with L-histidinium Dihydrogen Phosphate-Phosphoric Acid

Kamila Sałasińska [1,*], Maciej Celiński [1], Kamila Mizera [1,2], Mateusz Barczewski [3], Paweł Kozikowski [1], Michał K. Leszczyński [4,5] and Agata Domańska [6]

1 Central Institute for Labour Protection—National Research Institute, Department of Chemical, Biological and Aerosol Hazards, 00-701 Warsaw, Poland; macel@ciop.pl (M.C.); kamiz@ciop.pl (K.M.); pakoz@ciop.pl (P.K.)
2 Faculty of Materials Science and Engineering, Warsaw University of Technology, 02-507 Warsaw, Poland
3 Institute of Materials Technology, Poznan University of Technology, 61-138 Poznań, Poland; mateusz.barczewski@put.poznan.pl
4 Faculty of Chemistry, Warsaw University of Technology, 02-507 Warsaw, Poland; mleszczynski@ichf.edu.pl
5 Institute of Physical Chemistry, Polish Academy of Sciences, 01-224 Warsaw, Poland
6 Łukasiewicz Research Network—Institute for Engineering of Polymer Materials and Dyes, 87-100 Toruń, Poland; agata.domanska@impib.lukasiewicz.gov.pl
* Correspondence: kamila.salasinska@ciop.pl

Table S1. TG and DTG data of unmodified UP and resin with APP and LHP

Materials	UP	UP/ 10APP	UP/ 20APP	UP/ 30APP	UP/ 10LHP	UP/ 20LHP	UP/ 30LHP
$T_{5\%}$, °C	278	287	266	293	275	267	256
DTG1, °C; %/°C	231; 0.05	239; 0.04	184; 0.03	240; 0.03	228; 0.05	201; 0.05	230; 0.07
DTG2, °C; %/°C	-	326; 1.22	289; 0.21	323; 0.82	323; 0.68	320; 0.66	317; 0.54
DTG3, °C; %/°C	362; 1.00	407; 0.57	378; 0.57	389; 0.53	398; 0.68	403; 0.57	402; 0.49
DTG4, °C; %/°C	-	-	-	559; 0.27	-	-	-
DTG5, °C; %/°C	652; 0.02	668; 0.05	-	702; 0.06	-	-	-
DTG6, °C; %/°C	-	947; 0.02	874; 0.06	963; 0.02	896; 0.11	908; 0.11	913; 0.19
Residue in 1000 °C, %	1.4	2.6	3.7	2.5	2.4	4.5	4.3

Table S2. TG and DTG data of unmodified UP and resin with APP and LHP after immersion the samples in distilled water at 70 °C.

**Materials**	**UP ai**	**UP/30APP ai**	**UP/30LHP ai**
$W_{5\%}$, °C	276	263	257
DTG1, °C; %/°C	239; 0.05	234; 0.04	218; 0.06
DTG2, °C; %/°C	-	329; 0.76	315; 0.58
DTG3, °C; %/°C	359; 1.13	383; 0.52	401; 0.53
DTG4, °C; %/°C	595; 0.05	555; 0.22	-
DTG5, °C; %/°C		762; 0.04	
DTG6, °C; %/°C	-	944; 0.01	896; 0.14
Residue in 1000 °C, %	1.3	3.3	9.2
Leaching, %	1.13 (0.2) [a]	3.78 (0.5)	15.02 (1.4)

[a] The values in parentheses are the standard deviations.
